# Correction: Wan et al. Circadian Regulation of Alternative Splicing of Drought-Associated CIPK Genes in *Dendrobium catenatum* (Orchidaceae). *Int. J. Mol. Sci.* 2019, *20*, 688

**DOI:** 10.3390/ijms20163937

**Published:** 2019-08-13

**Authors:** Xiao Wan, Long-Hai Zou, Bao-Qiang Zheng, Yan Wang

**Affiliations:** Research Institute of Forestry, State Key Laboratory of Tree Genetics and Breeding, Chinese Academy of Forestry, Beijing 100091, China

We would like to submit several corrections to our published paper “Circadian Regulation of Alternative Splicing of Drought-Associated CIPK Genes in *Dendrobium catenatum* (Orchidaceae)” (doi: 10.3390/ijms20030688) in the *International Journal of Molecular Sciences* [1]. The IDs of CIPK genes in *Dendrobium catenatum* were initially named according to their positions at genome scaffolds. After the first revision, one of the reviewers suggested that the IDs should be renamed according to gene homology; thus, we changed these IDs. Unfortunately, because there were very many that needed to be changed, some numerical problems were still neglected. Hence, here we describe the corrections below.

In the abstract, “Expression patterns of CIPK family genes in different tissues and in response to either drought or cold stresses suggested *DcaCIPK11* may be associated with signal transduction and energy metabolism. *DcaCIPK9*, -*14*, and -*16* are predicted to play critical roles during drought treatment specifically. Furthermore, transcript expression abundances of *DcaCIPK16* showed polar opposites during day and night. Whether under drought treatment or not, *DcaCIPK16* tended to emphatically express transcript1 during the day and transcript3 at night. This implied that expression of the transcripts might be regulated by circadian rhythm. qRT-PCR analysis also indicated that *DcaCIPK3*, -*8*, and -*20* were strongly influenced by circadian rhythmicity.” should be corrected to “Expression patterns of CIPK family genes in different tissues and in response to either drought or cold stresses suggested *DcaCIPK14* may be associated with signal transduction and energy metabolism. *DcaCIPK3*, -*12*, and -*16* are predicted to play critical roles during drought treatment specifically. Furthermore, transcript expression abundances of *DcaCIPK3* showed polar opposites during the day and night. Whether under drought treatment or not, *DcaCIPK3* tended to emphatically express transcript5 during the day and transcript2 at night. This implied that expression of the transcripts might be regulated by circadian rhythm. qRT-PCR analysis also indicated that *DcaCIPK1*, -*7*, and -*15* were strongly influenced by circadian rhythmicity”.

In Section 2.5, “*Expression of D. catenatum CIPK Genes under Abiotic Stress*”, the sentence “CIPK20 and -21 were significantly fluctuant under drought and cold treatment as compared with normal conditions, respectively.” should be corrected to “Twenty and Twenty-one CIPK genes were significantly fluctuant under drought and cold treatment as compared with normal conditions, respectively”.

In Section 2.6, “*Alternative Splicing Analysis of CIPK Members under Drought Stress*”, the second paragraph, “and the two main transcripts (tanscript1 and transcript3) in the latter gene had a mutually day-and-night reversed expression (Figure 6B)” should be corrected to “and the two main transcripts (tanscript5 and transcript 2) in the latter gene had a mutually day-and-night reversed expression (Figure 6B)”.

In Section 3.3, “*Circadian Rhythm and Drought Stress Both Influence Alternative Splicing of CIPK Members*”, “Under drought treatment, the expression levels of different transcripts suggested that the most highly expressed transcript variant of *DcaCIPK3* tended to be transcript1 during the daytime and transcript3 at nighttime. The moist treatment had a negative effect on the sum expression abundance of various transcripts, but did not change the routine preference of transcript1 during the day and transcript3 at night, respectively.” should be corrected to “Under drought treatment, the expression levels of different transcripts suggested that the most highly expressed transcript variant of *DcaCIPK3* tended to be transcript5 during the daytime and transcript2 at nighttime. The moist treatment had a negative effect on the sum expression abundance of various transcripts, but did not change the routine preference of transcript5 during the day and transcript2 at night, respectively.”

These changes have no material impact on the conclusions of our paper. The authors apologize for any inconvenience caused to the readers by these changes.
