# Supplementary figures and images for: Circadian Regulation of Alternative Splicing of Drought-Associated CIPK Genes in Dendrobium catenatum (Orchidaceae)
Source: Int J Mol Sci. 2019 Feb 5;20(3):688. doi: 10.3390/ijms20030688 (PMC6386984; doi:10.3390/ijms20030688)

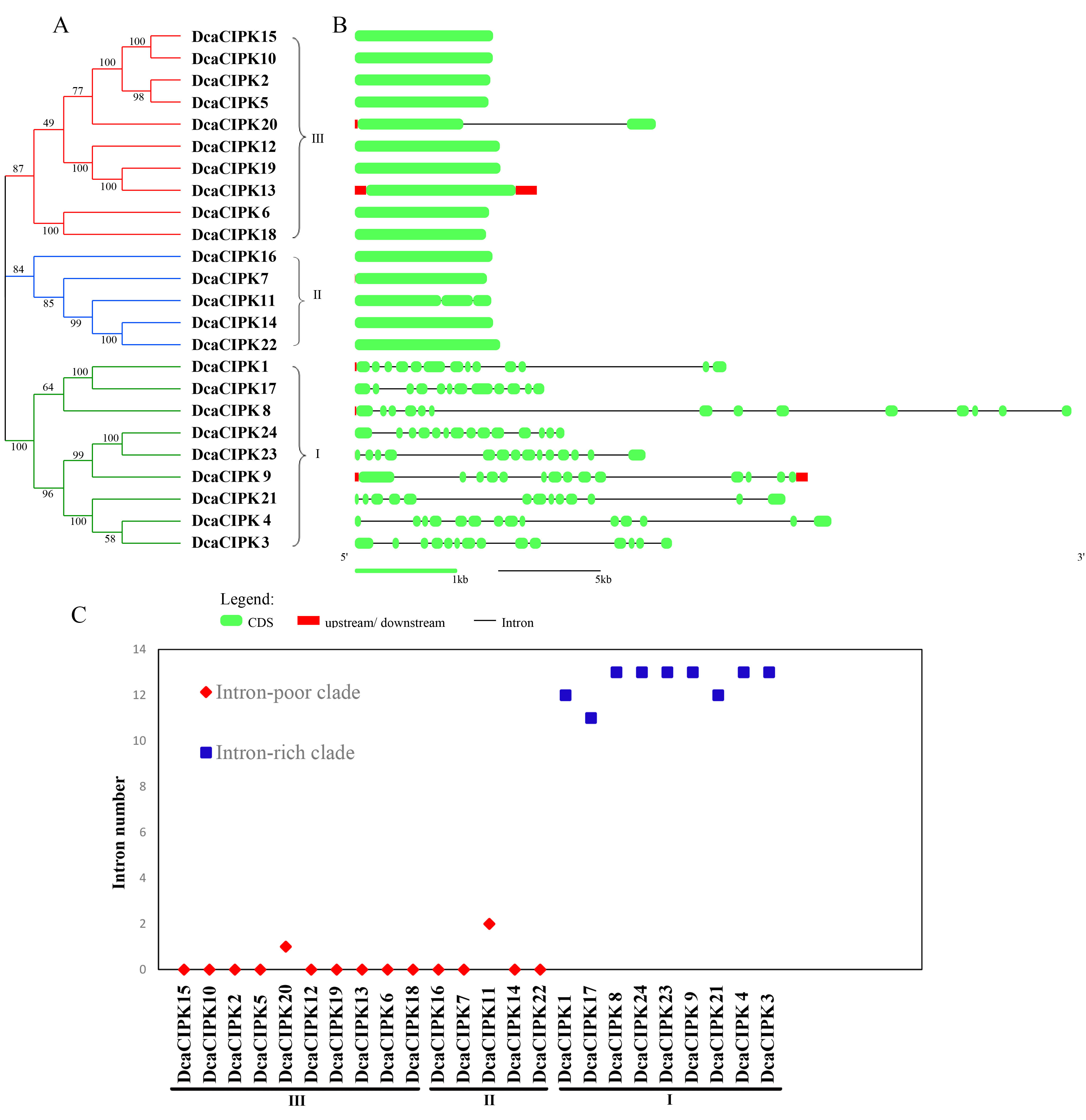

Supplement: Supplementary file 1 [file ijms-20-00688-s001.zip › supplementary file v2/Figure 1.jpg]

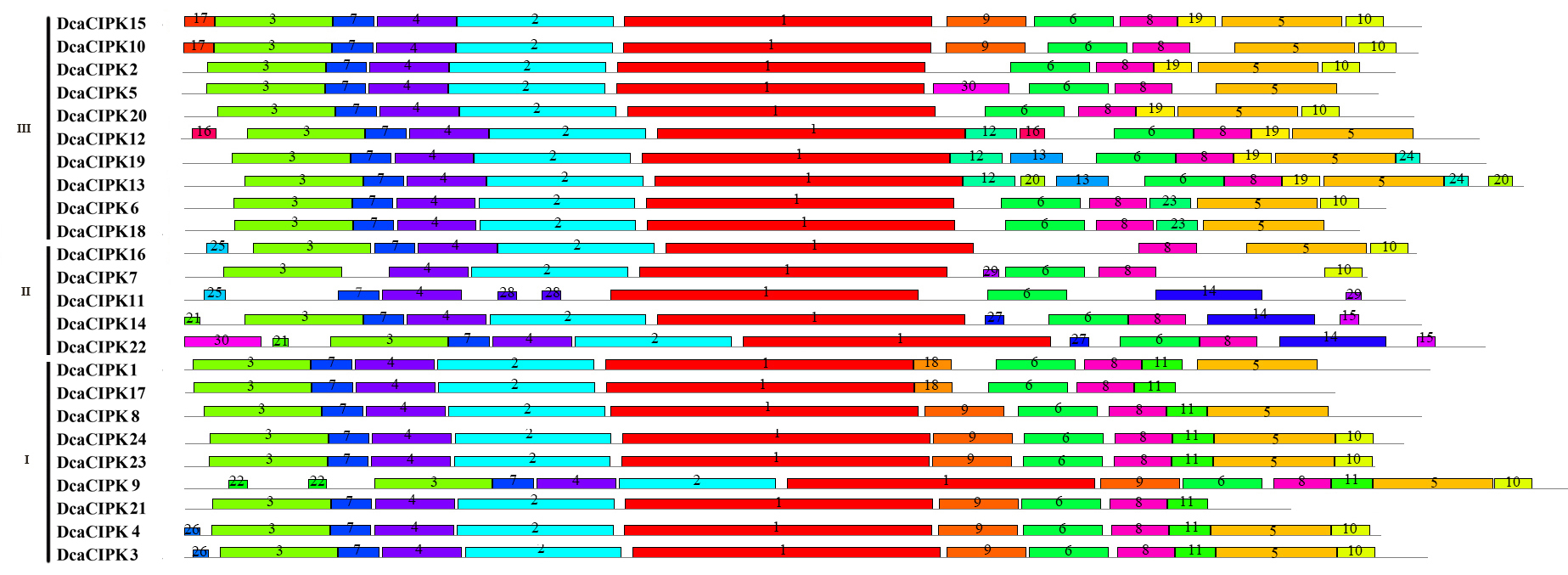

Supplement: Supplementary file 1 [file ijms-20-00688-s001.zip › supplementary file v2/Figure 2.jpg]

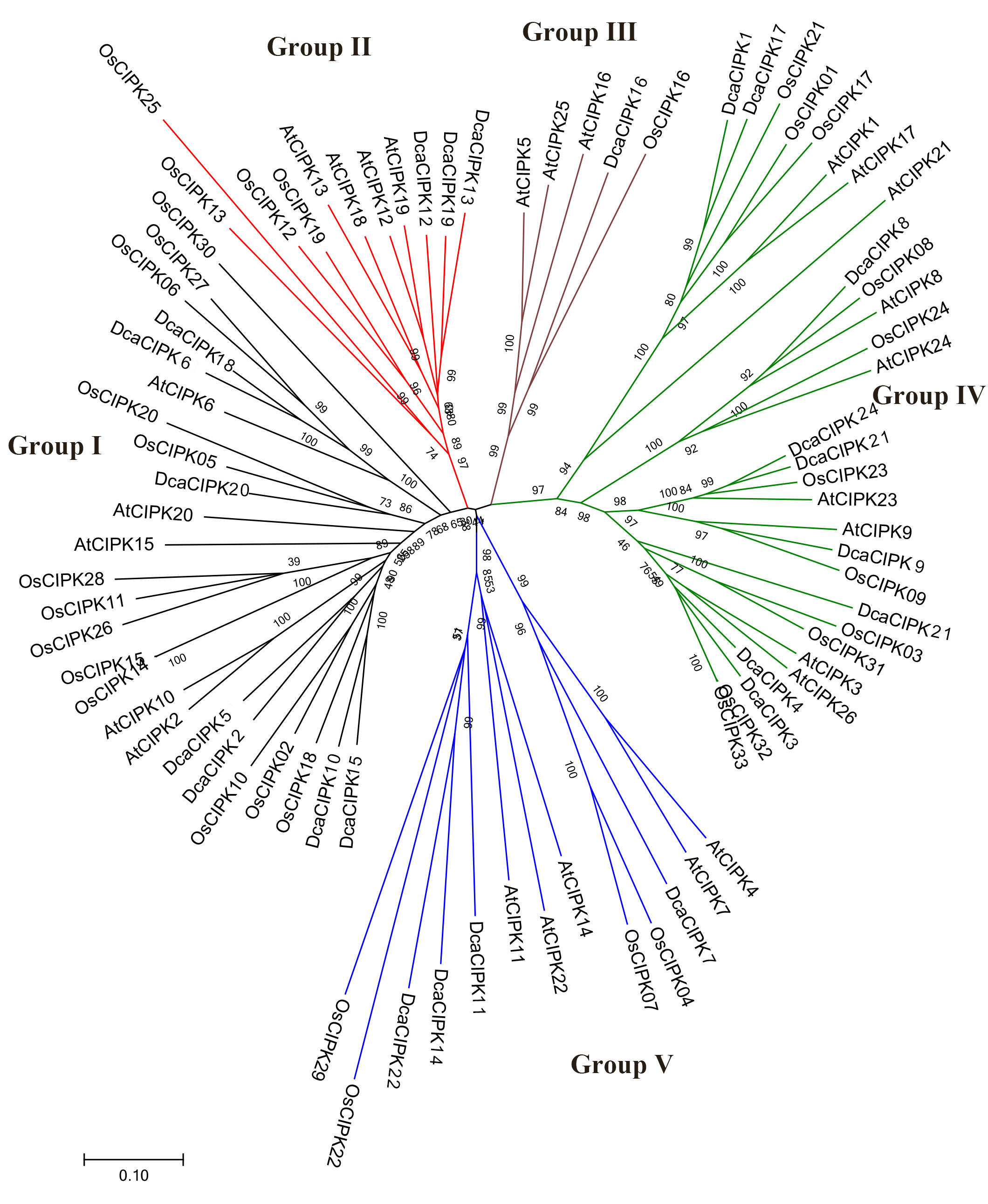

Supplement: Supplementary file 1 [file ijms-20-00688-s001.zip › supplementary file v2/Figure 3.jpg]

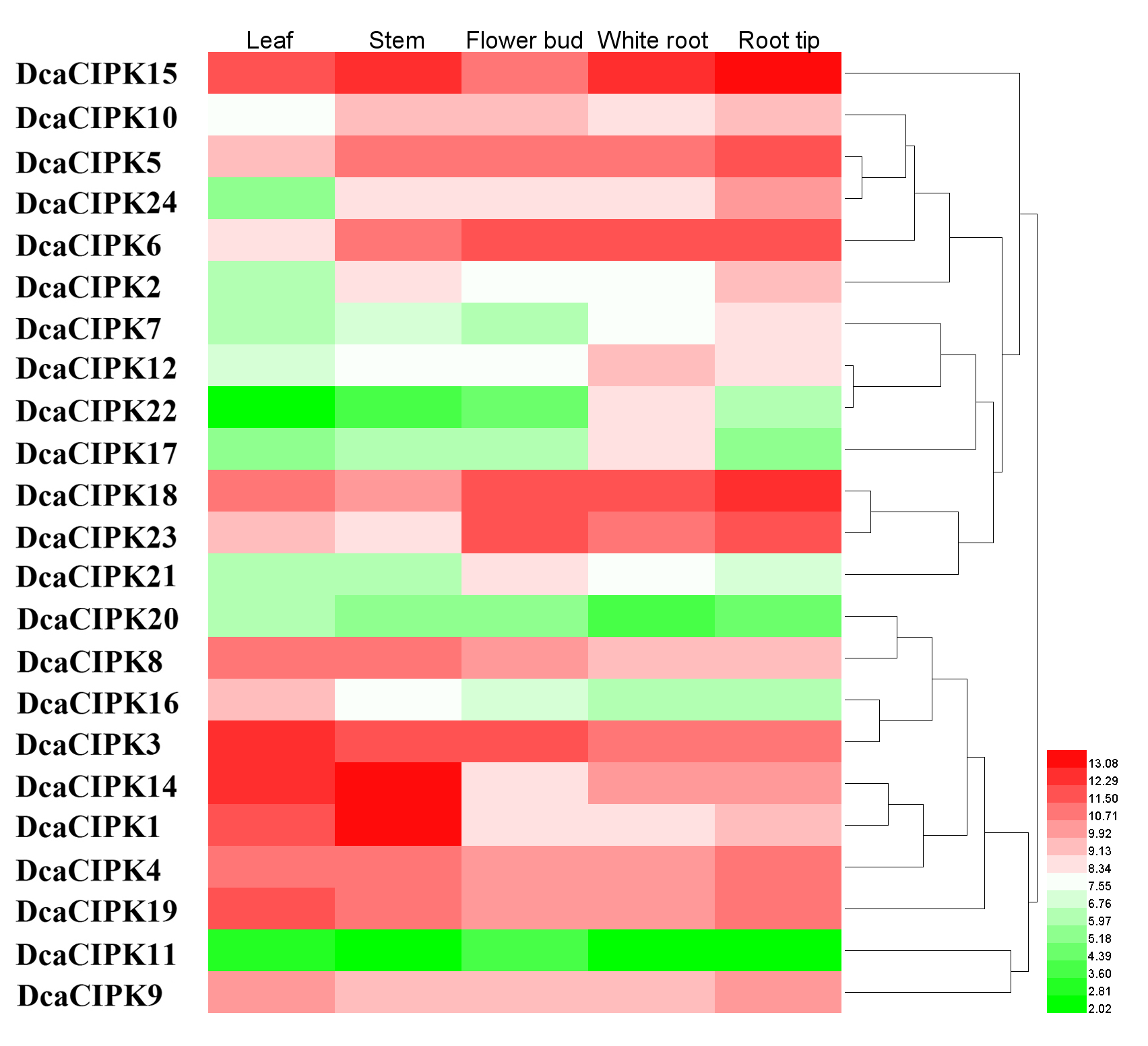

Supplement: Supplementary file 1 [file ijms-20-00688-s001.zip › supplementary file v2/Figure 4.tif]

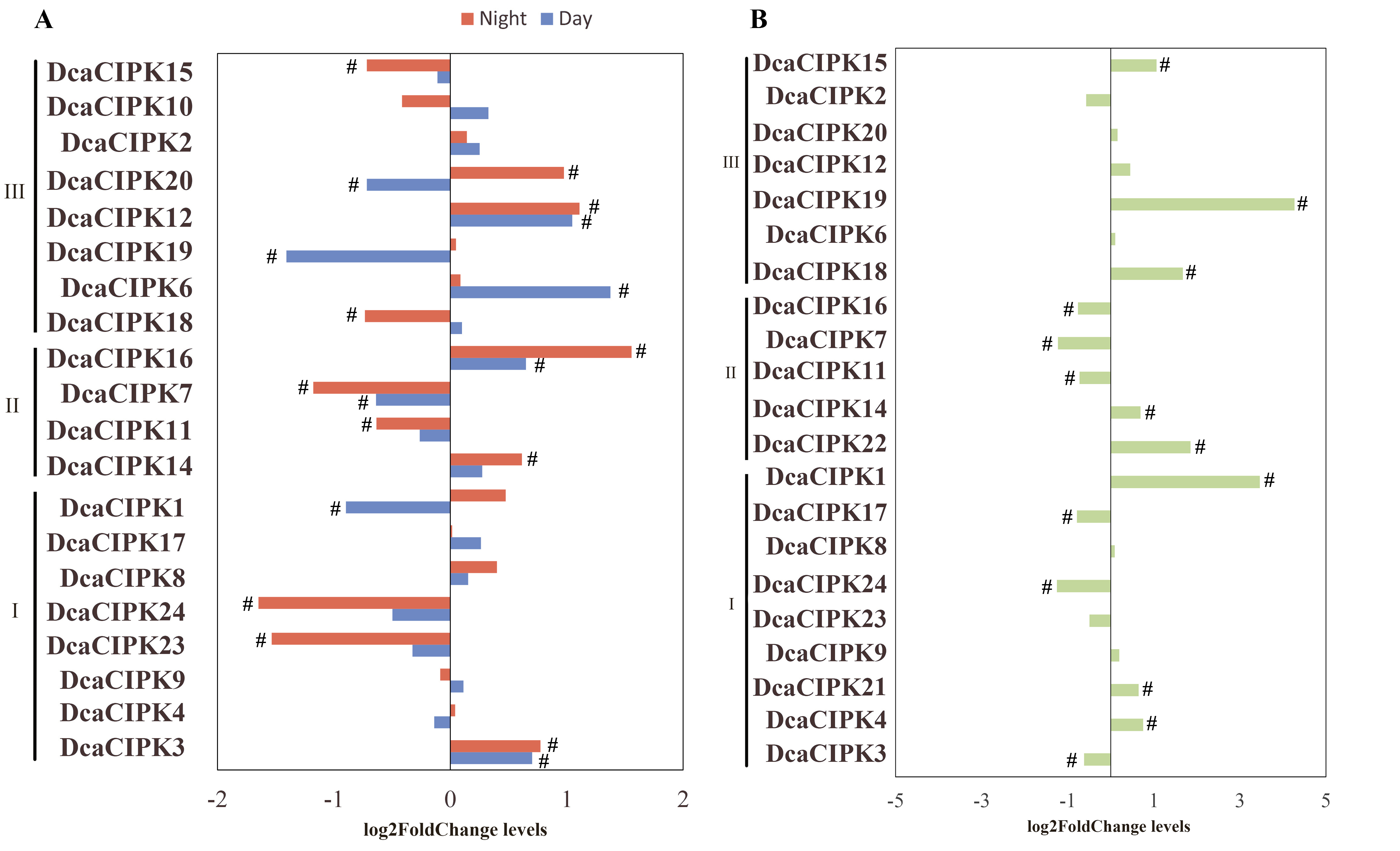

Supplement: Supplementary file 1 [file ijms-20-00688-s001.zip › supplementary file v2/Figure 5.tif]

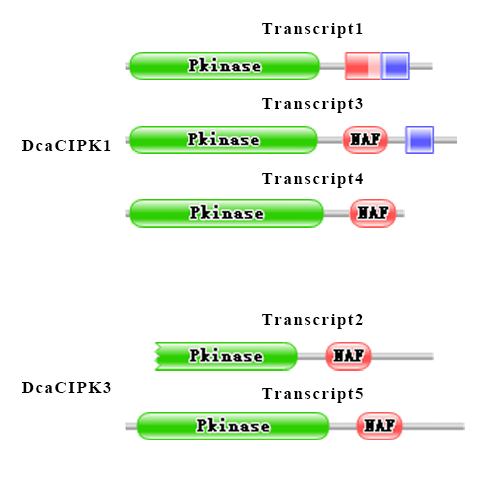

Supplement: Supplementary file 1 [file ijms-20-00688-s001.zip › supplementary file v2/Figure 7.tif]

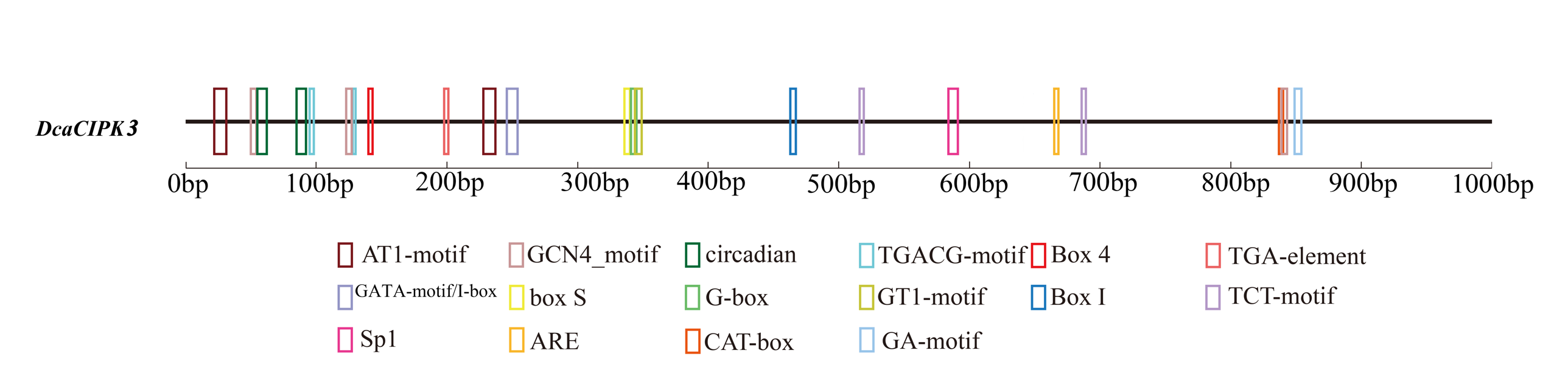

Supplement: Supplementary file 1 [file ijms-20-00688-s001.zip › supplementary file v2/Figure 8.jpg]

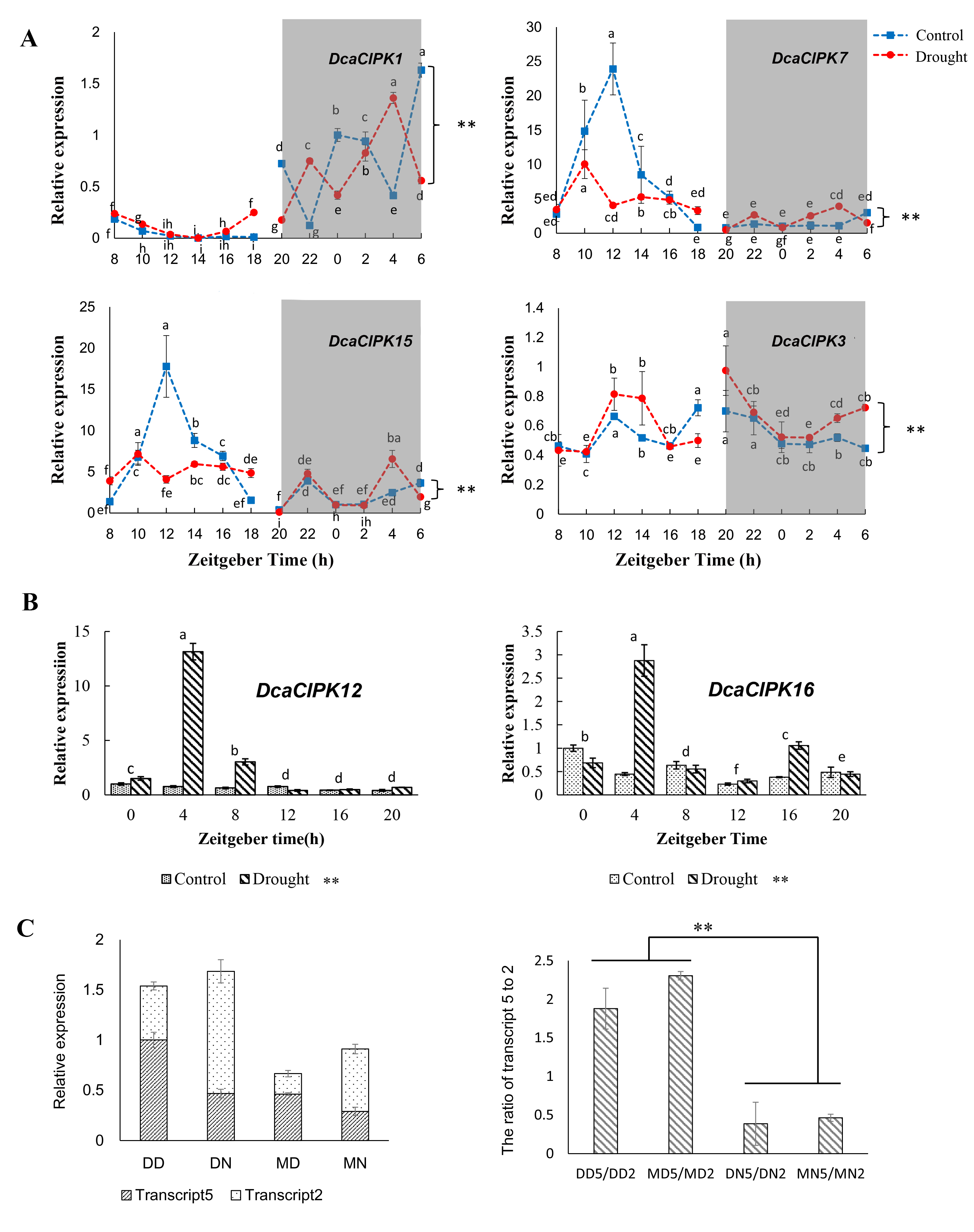

Supplement: Supplementary file 1 [file ijms-20-00688-s001.zip › supplementary file v2/Figure 9.tif]
